# External Subretinal Fluid Drainage in Scleral Buckling: Before Versus after Cryotherapy and Buckle Placement, A Pilot Study

**DOI:** 10.3390/life13020284

**Published:** 2023-01-19

**Authors:** Tessnim R. Ahmad, Gregory J. Bever, Jay M. Stewart

**Affiliations:** 1 Department of Ophthalmology, University of California, San Francisco, CA 94158, USA; 2 Department of Ophthalmology, Zuckerberg San Francisco General Hospital and Trauma Center, San Francisco, CA 94110, USA

**Keywords:** scleral buckle, needle drainage, subretinal fluid, technique, cryotherapy, retinal detachment, rhegmatogenous

## Abstract

In this retrospective comparative case series at a teaching hospital, we reviewed adult patients with rhegmatogenous retinal detachment who underwent scleral buckling surgery with external drainage of subretinal fluid performed before versus after placement of the scleral buckle. Eight eyes in each group were roughly matched for age, sex, baseline visual acuity (VA), and detachment characteristics. The complication rate was 0% for the “before” group and 37% for the “after” group (*p* = 0.100). In the “after” group, two eyes (25%) developed iatrogenic retinal holes and one eye (12%) developed self-limited subretinal hemorrhage during external needle drainage. The duration of surgery was significantly shorter for the “before” group (mean 89 ± 16 min) compared to the “after” group (118 ± 20 min) (*p* = 0.008). The primary anatomic success rate was 100% for the “before” group and 75% for the “after” group (*p* = 0.233). Final VA was not significantly different between the groups or from baseline. In conclusion, while limited by our small sample size, this pilot study suggests that drainage of subretinal fluid before scleral buckle placement may be safer and more efficient compared to draining after buckle placement. Initial drainage may facilitate retinochoroid apposition to allow targeted cryopexy and precise buckle placement.

## 1. Introduction

Successful treatment of rhegmatogenous retinal detachment (RRD) requires closure of all retinal breaks, a principle first demonstrated by Jules Gonin in 1930 [1]. Gonin’s original procedure used needle thermocautery to seal retinal breaks. Subretinal fluid (SRF) drained as the needle was withdrawn [1]. In 1949, Ernst Custodis developed a polyviol exoplant to encircle the eyeball and induce functional closure of breaks [2]. Such scleral buckling has grown in favor and remains the preferred treatment for phakic patients without a posterior vitreous detachment (PVD) [3]. 

A typical scleral buckling procedure involves cryotherapy to retinal breaks followed by application of the buckle. The need for drainage of SRF has posed controversy [4,5]. Factors influencing the decision to drain include the size and location of the break(s), the height of detachment, and the appearance of the retina after buckle placement [6]. Drainage prior to cryotherapy or buckle placement is not routinely performed.

Conventional drainage of SRF involves sclerotomy and external diathermy [7]. An alternative approach was first introduced by Steve Charles in 1985 using oblique insertion of a 25-gauge needle under direct visualization with indirect ophthalmoscopy [8]. Modifications have since been described, including incorporation of chandelier endo-illumination [9] and a wide-angle viewing system to improve visualization [10], use of a guarded needle to prevent overpenetration [11], drainage under a tightened buckle to reduce the risk of hemorrhage [12], perpendicular insertion of a hub needle [13], use of a suture needle with [14] or without [15,16] continuous monitoring, use of a cold diathermy pin [17], and cannula-controlled drainage [18].

External drainage of subretinal fluid has often been performed after scleral buckle placement, out of concern that low intraocular pressure after drainage could make scleral buckle placement more difficult. In this small retrospective comparative study, we compare cases in which drainage was performed before versus after scleral buckle placement. We hypothesize that initial drainage before buckle placement could facilitate retinochoroid apposition and therefore improve the accuracy of cryotherapy and buckle placement. In draining before buckle placement, we also incorporate a less commonly used external needle drainage technique without simultaneous visualization of the fundus. We compare the outcomes between patients treated with these two approaches.

## 2. Materials and Methods

### 2.1. Study Design

This is a retrospective comparative study of adult patients with RRD who underwent scleral buckling by a team consisting of one attending vitreoretinal surgeon and one vitreoretinal surgery fellow. The same attending and fellow team performed all surgeries in this study. Institutional Review Board approval was obtained. Inclusion criteria included phakic lens status and the absence of PVD by optical coherence tomography (OCT; no separation of the posterior hyaloid from the inner surface of the retina). Patients were treated with drainage before buckle placement (the “before” approach) beginning February 2019. An equal number of similar patients treated with the “after” approach were retrospectively and consecutively selected. Variables reviewed in the medical record included past medical and ocular history; visual acuity (VA); detachment and break(s) features; surgical technique; and complications. Primary anatomic success was defined as retinal reattachment without any secondary retina-affecting surgery. The OCT was performed preoperatively and at postoperative week 1 and month 1 for all patients.

### 2.2. Drainage before Buckle Placement: Operative Technique

**Figure 1:**
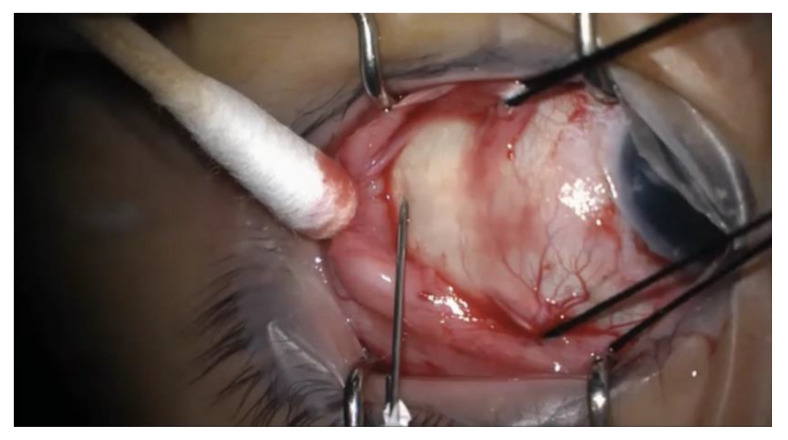
Intraoperative photograph of the needle insertion for external drainage of subretinal fluid. A 25-gauge needle on a tuberculin syringe is introduced transclerally at an oblique angle into the subretinal space. The needle is removed immediately after entering the sclera, and subretinal fluid is expressed by depressing the posterior lip of the wound using a cotton swab.

Westcott scissors were used to create a conjunctival peritomy in proportion to the anticipated circumference of the buckle, and the appropriate rectus muscle(s) were isolated. In conjunction with the noncontact viewing system of the operating microscope, a light pipe or chandelier via a trocar-cannula system was used to inspect the retina for areas of detachment and breaks. A 25-gauge needle on a tuberculin (TB) syringe was introduced at an oblique angle into the subretinal space, transclerally, in the area that was to be covered by the buckle. See Figure 1 for an intraoperative photograph of the needle insertion. The needle was removed immediately after entering the sclera. This stab incision was done without simultaneous visualization of the fundus. The SRF was expressed from the drainage site by depressing the posterior lip of the wound using a cotton swab. Careful inspection confirmed reattachment of the retina, and cryotherapy was applied in the location of the break(s). Nylon sutures (5-0), optionally placed before or after drainage of the subretinal fluid, were passed through the partial thickness of the sclera in a horizontal mattress fashion at 1 mm and 7 mm to 10 mm (depending on the buckle size) posterior to the level of the muscle insertion. The scleral buckle was subsequently passed beneath the sutures and the rectus muscle(s), with the two free ends joined with a sleeve for encircling buckles. For segmental buckles, the ends were left free with either one or two horizontal mattress sutures passed in each intermuscular quadrant containing retinal pathology. An anterior chamber paracentesis or injection of a sterile balanced salt solution (BSS) through the pars plana cannula was performed with a 30-gauge needle if needed to restore normal pressure. The buckle contour on the retina was visualized to ensure adequate support of the break, with adjustment via suture removal and replacement if necessary. Optic nerve perfusion was confirmed on ophthalmodynamometry. A representative surgical video demonstrates the surgical technique for one of the patients in this case series (accessible via the following link: https://tinyurl.com/5csb8php).

**Surgical video**: Intraoperative video of the restructured surgical approach to scleral buckling. Steps include stab incision drainage followed by cryotherapy and then buckle placement. Accessible via the following link: https://ucsf.box.com/s/uz1fmvly14t2vsixpmf04lk5pnsr74lv.

## 3. Results

Eight eyes of seven patients were treated with the “before” procedure. Eight eyes of eight patients were retrospectively and consecutively identified to demonstrate the “after” procedure (from September 2016 to February 2019). The most common “after” approach (37%) involved cryotherapy followed by buckle placement then needle drainage. Other sequences included cryotherapy-needle drainage-buckle placement (25%), buckle placement-cryotherapy-needle drainage (12%), and buckle placement-needle drainage-cryotherapy (12%). Needle drainage was performed under visualization and involved transcleral insertion of a 25-gauge short needle on a TB syringe.

The eight eyes in each group were roughly matched for age (*p* = 0.133), sex (*p* = 1.000), and detachment characteristics. Median preoperative VA (Snellen) was 20/30 for the restructured group and 20/50 for the traditional group (*p* = 0.189). The median number of detached clock hours was three for the “before” group and four for the “after” group (*p* = 0.221). Most detachments were macula on (75% in the “before” group, 62% in the “before” group; *p* = 1.000). See Table 1 for clinical and surgical characteristics by group.

There were no complications in the “before” group. Two eyes (25%) in the “after” group developed iatrogenic retinal holes during external needle drainage which were successfully repaired with cryotherapy in one case and cryotherapy and a segmental buckle in the second case. One eye in the “after” group (12%) developed self-limited subretinal hemorrhage following external needle drainage. The duration of surgery was significantly shorter for the “before” group (mean 89 ± 16 min) compared to the “after” group (118 ± 20 min) (*p* = 0.008). All eyes in the “before” group achieved primary anatomic success at a mean follow-up of nine months (range: 4–21). The primary anatomic success rate for the “after” group was 75% at a mean follow-up of 14 months (range: 2–24). One patient required buckle revision for progression of subretinal fluid, and the second patient required vitrectomy for recurrent detachment, both at postoperative month 1. Final VA was 20/25 for the “before” group and 20/30 for the “after” group (*p* = 0.202), which were not significantly different from baseline for either group (*p* = 0.585 and *p* = 0.501, respectively). Table 2 provides detailed characteristics for the eyes in each group.

## 4. Discussion

We demonstrate an alternative scleral buckling procedure involving initial external needle drainage followed by cryotherapy and then buckle placement. Our small comparative study suggests the stab incision approach may have a better safety profile compared to traditional needle drainage techniques, without the need for simultaneous visualization of the fundus. Initial drainage before buckle placement may also improve retinochoroid apposition to allow targeted cryopexy and precise buckle placement with lower risk of recurrent detachment. All patients treated with the “before” approach achieved primary anatomic success, compared to 75% of those treated with “after” approaches, similar to previous reports [19,20]. Likely related to these differences, the duration of surgery was significantly shorter for patients treated with the “before” approach.

The drainage technique presented herein involves oblique introduction of a 25-gauge needle into the subretinal space transclerally. We have found that a brief entry of just the tip of the needle, followed by its withdrawal, creates a sclerotomy and choroidotomy of sufficient size to allow safe and reliable drainage of SRF, even the relatively viscous fluid associated with chronic retinal detachments. The creation of an iatrogenic retinal hole or retinal incarceration can be avoided by selecting a drainage site in an area with a relatively greater height of detachment, keeping the needle entry oblique (and adjusting the degree of the angle to the height of detachment), and allowing only a very short segment of the needle tip to enter the subretinal space. In this study, retinal holes occurred in 25% of the eyes treated with the “after” technique. This is higher than previous reports demonstrating a less than 2% incidence [13,14,15,16,17,21,22]. The technical difficulty of performing needle drainage with simultaneous visualization, especially with fellow surgeon involvement at our institution, may have contributed to this high complication rate. Regardless, since these complications are generally rare, larger studies are required to establish the safety of the “before” procedure.

Another potential concern could be the introduction of complications related to the relative hypotony induced by performing drainage at the beginning of the procedure, namely intraocular hemorrhage or difficulty passing scleral sutures. Neither materialized as a barrier. Rates of subretinal hemorrhage vary widely between techniques, from 0 to 28 percent, though direct comparison is limited by differences in case numbers and patient populations [12,13,14,15,16,17,21,22]. We observed one case of subretinal hemorrhage (12%) in the “after” group and none in the “before” group. We do not consider the present technique to have a different risk profile than a traditional approach in which drainage is performed after buckle placement but before tightening of the scleral sutures around the buckle. In either case, there is a brief period during which the pressure is low immediately after drainage. In both approaches, the next step is to visualize the retina and assess the amount of remaining SRF, at which point external pressure could be applied to tamponade any bleeding. Additionally, this technique already employs intense external pressure to provide initial tamponade if needed. Regarding passing sutures after drainage, the suture need only take a short course through the sclera to provide sufficient strength to hold the buckle element in place and provide the necessary imbrication to support retinal breaks. Additionally, BSS can be injected intravitreally to reinflate the eye, as was done in one case. Finally, we note that sutures can be placed before drainage, while the eye is still normotensive; this still allows more precise cryopexy to the attached retina after subretinal fluid drainage but risks requiring re-suturing or moving the buckle if the sutures do not turn out to be in the precise location needed for optimal support of the retinal breaks once the buckle is placed.

Our technique utilizes endo-illumination and the noncontact viewing system of the operating microscope for intraoperative visualization of the retina. This method has previously been described by various authors [9,22,23]. Although we have found it to be useful, it is not essential for the modifications we propose to scleral buckling surgery. Visualization with the binocular indirect ophthalmoscope and a handheld lens is also suitable and compatible with the modifications we propose. A major limitation of this study was the small sample size. We initiated the “before” technique in February 2019 and limited case selection to the same vitreoretinal attending-fellow pair. We retrospectively selected the same number of cases (performed by the same surgical pair) using the “after” technique. Future study with a larger number of patients is warranted.

## 5. Conclusions

This pilot study suggests that it may be reasonable to drain subretinal fluid early in the scleral buckle procedure, using a less commonly used external needle drainage technique via a stab incision followed by cryotherapy and buckle placement. While limited by the small sample size and retrospective design, the present study suggests that this technique may increase the safety, efficiency, and effectiveness of scleral buckling.

## Figures and Tables

**Table 1:** Characteristics of patients undergoing scleral buckling.

	Drainage before Buckle Placement (*n* = 8)	Drainage after Buckle Placement (*n* = 8)	*p*-Value
Sex (% female)	62%	50%	1.000
Age (years) (mean ± SD)	36 ± 11	29 ± 7	0.133
Baseline VA (logMAR) (mean ± SD)	0.14 ± 0.21	0.37 ± 0.44	0.189
Number of detachment clock hours (mean ± SD)	3.1 ± 1.2	4.2 ± 2.2	0.221
Number of breaks in detachment (mean ± SD)	1.9 ± 1.0	2 ± 1.2	0.798
Macula status (% on)	75%	62%	1.000
Complication rate	0%	37%	0.100
Surgery duration (minutes) (mean ± SD)	89 ± 16	118 ± 20	0.008 *
Primary anatomic success rate	100%	75%	0.233
Final VA (logMAR) (mean ± SD)	0.087 ± 0.13	0.24 ± 0.30	0.202
Final anatomic success rate	100%	100%	1.000
Follow-up time (months) (mean ± SD)	9 ± 6	14 ± 10	0.158

* *p* < 0.050, SD: standard deviation, VA: visual acuity, logMAR: logarithm of minimum angle of resolution.

**Table 2:** Detailed characteristics of patients undergoing surgery with subretinal fluid drainage before vs. after buckle placement.

Patient	Group/Eye	Sex	Age	Baseline VA	Detachment Clock Hours	Number of Breaks in Detachment	Macula Status	Buckle Extent	Operative Sequence	Intraoperative Complications	Surgery Duration (Minutes)	Final VA	Primary Anatomic Success	Follow-Up (Months)
1	Before 1	Male	28	20/20	8:00 to 11:00	1	On	Encircling	Drainage-cryotherapy-buckle	None	107	20/25	Yes	14
2	Before 2	Male	42	20/20	9:00 to 12:00	1	On	Segmental	Drainage-cryotherapy-buckle	None	77	20/20	Yes	6
3	Before 3	Male	23	20/40	12:30 to 3:00	3	Off	Segmental	Drainage-cryotherapy-buckle	None	73	20/40	Yes	10
4	Before 4	Female	35	20/20	6:00 to 9:00	3	On	Segmental	Drainage-cryotherapy-buckle	None	95	20/20	Yes	7
5	Before 5	Female	31	20/20	3:00 to 7:30	3	On	Segmental	Drainage-cryotherapy-buckle	None	96	20/20	Yes	5
6	Before 6	Female	31	20/25	5:00 to 7:00	2	On	Segmental	Drainage-cryotherapy-buckle	None	62	20/20	Yes	4
7	Before 7	Female	55	20/25	9:30 to 11:00	1	On	Segmental	Drainage-cryotherapy-buckle	None	105	20/20	Yes	21
8	Before 8	Female	47	20/80	3:00 to 8:00	1	Off	Segmental	Drainage-cryotherapy-buckle	None	97	20/40	Yes	4
9	After 1	Male	21	20/80	12:00 to 7:00	2	Off	Encircling	Cryotherapy-drainage-buckle	None	120	20/60	Yes	24
10	After 2	Female	24	20/50	5:30 to 9:00	1	On	Segmental	Buckle-drainage-cryotherapy	Iatrogenic retinal hole *	110	20/40	Yes	10
11	After 3	Male	26	20/20	1:30 to 6:00	3	On	Segmental	Buckle-cryotherapy-drainage	Iatrogenic retinal hole †	158	20/25	Yes	2
12	After 4	Female	24	20/40	4:00 to 10:00	1	Off	Segmental	Cryotherapy-buckle-drainage	None	122	20/20	Yes	5
13	After 5	Male	43	20/20	7:00 to 7:30	1	On	Segmental	Cryotherapy-buckle-drainage	None	93	20/20	No ‡	23
14	After 6	Female	35	20/400	4:00 to 8:00	2	Off	Segmental	Cryotherapy-drainage-buckle	None	110	20/150	Yes	23
15	After 7	Male	30	20/50	1:00 to 7:00	3	On	Segmental	Cryotherapy-buckle-drainage	Subretinal hemorrhage ❡	116	20/25	No §	17
16	After 8	Female	31	20/20	7:00 to 9:00	3	On	Segmental	Cryotherapy-buckle-drainage	None	-	20/25	Yes	10

VA: visual acuity, Snellen. * Occurred during external needle drainage and was successfully repaired with cryotherapy. † Occurred during external needle drainage and was successfully repaired with cryotherapy and a radial buckle. ‡ Buckle revision was required at postoperative month 1 for progression of subretinal fluid leading to inadequate break support. Note that drainage was not performed in the initial repair. § Redetached at approximately postoperative month 1. This was successfully repaired with pars plana vitrectomy. ❡ There was localized subretinal bleeding following external needle drainage. This was well outside the macula and did not progress.

## Data Availability

The datasets generated during the current study are not publicly available as they pertain to individual patients but are available from the corresponding author on reasonable request.
